# Dopamine-NMDA interactions and relevance to gamma band synchrony in schizophrenia

**DOI:** 10.1186/1471-2202-14-S1-P218

**Published:** 2013-07-08

**Authors:** Kübra Kömek, George B Ermentrout, Raymond Y Cho

**Affiliations:** 1 Center for the Neural Basis of Cognition, Carnegie Mellon University, Pittsburgh, PA, 15213, USA; 2 Department of Mathematics, University of Pittsburgh, Pittsburgh, PA, 15213, USA; 3 Department of Psychiatry, University of Pittsburgh, Pittsburgh, PA, 15213, USA

## 

An increasing body of behavioral and physiological evidence shows that cortical activity operates optimally within a limited range of dopamine (DA) transmission within a broader inverted-U shaped relationship [1]. However, the exact mechanism through which DA leads to such non-monotonic modulation of network activity needs further investigation. Our recent work suggests that DA's modulation of potassium currents in the fast-spiking interneurons could be a potential mechanism underlying this inverted-U relationship [2]. Here we propose that another potential mechanism could contribute in the form of interactions between DA D1 receptors and NMDA conductance in the cortex. This has been an area of interest for many reasons including its relevance to the etiology of schizophrenia with deficits in both NMDA-mediated glutamate and DA transmission. Studies examining the relationship between DA and NMDA conductance in the cortex revealed that DA D1 receptors enhance NMDA responses [3-5].

In the current study, we incorporated DA's modulation of NMDA conductance at a network level and analyzed whether DA's modulation of NMDA currents could lead to an inverted-U shaped relationship with cortical activity. With this motivation, we simulated a neural network with 200 excitatory and 40 inhibitory quadratic integrate-and-fire neurons coupled with biologically realistic probabilities. In the simulated network, the effects of DA were implemented through varying the NMDA conductances in various combinations of synaptic types including excitatory-excitatory and excitatory-inhibitory. The network synchronization was analyzed through examining gamma band power in the local field potential. Parametrically varying the NMDA conductance revealed an inverted-U shaped relationship, with lower gamma band power at both low and high conductance levels and optimal synchronization occurring at intermediate conductance levels. Our findings reveal that DA's modulation of NMDA currents at the single cell level gives rise to a non-monotonic relationship between cortical gamma band synchrony and DA levels. These results together with those of [2] suggest that DA's modulation of potassium currents as well as NMDA-mediated currents may be complementary or synergistic mechanisms that give rise to the inverted-U shaped pattern in cortical activity across dopamine levels.
